# Healthcare Professionals’ Perspective of the Implementation of Age-Friendly Care 4Ms Rounding: A Case Study

**DOI:** 10.1093/geroni/igaf122.326

**Published:** 2025-12-31

**Authors:** Erin Kitt-Lewis, Zahra Ezzi

**Affiliations:** Penn State, State College, Pennsylvania, United States; Penn State Ross and Carol Nese College of Nursing, University Park, Pennsylvania, United States

## Abstract

The Age-Friendly Care Health System (AFHS) initiative, launched by the Institute for Healthcare Improvement in collaboration with John A. Hartford Foundation, focuses on providing care that addresses the unique needs of older adults. The 4Ms Framework – What Matters, Medications, Mentation, and Mobility – provides a model to ensure that care for older adults is comprehensive and focused on their goals and priorities. AHFS 4Ms rounding process was implemented into one long-term care facility by this PI and an interdisciplinary team of nursing home staff. The purpose of this case study was to explore the perspectives of the interdisciplinary staff (e.g., physician, nurse practitioner, social worker, registered nurse, licensed practical nurse, nursing home assistant administrator) that participated in the implementation of the Age-Friendly Care 4Ms Rounding with residents. Interdisciplinary staff were interviewed using a semi-structured interview guide. Audio recorded interviews were transcribed, verified, and de-identified. Thematic analysis was conducted. Findings revealed healthcare professionals’ perspective of the implementation of Age-Friendly Care 4Ms rounding. Themes include the impact of 4Ms rounding; factors that support and hinder the integration of the rounding process; and recommendation for improving and sustaining rounding for better integration into this care setting and potential future care settings. These findings will be used to refine the rounding process and create sustainable changes to procedures and practices in this nursing home. In addition, a template will be created so that additional long-term care facilities can replicate this process of implementing Age-Friendly Care 4Ms rounding.

